# The need for improved integration of psychosocial and supportive care in cancer: a qualitative study of Australian patient perspectives

**DOI:** 10.1007/s00520-025-09593-5

**Published:** 2025-05-30

**Authors:** Clare Lynex, Drew Meehan, Kate Whittaker, Tanya Buchanan, Megan Varlow

**Affiliations:** 1 Cancer Council Australia, Sydney, Australia; 2 School of Health and Society, Faculty of the Arts, Social Sciences and Humanities, University of Wollongong, Wollongong, NSW 2522 Australia

**Keywords:** Cancer, Mental health, Peer support, Psycho-oncology, Psychosocial, Supportive care

## Abstract

**Purpose:**

It is well established that people diagnosed with cancer often experience poor mental health, yet there are still inequities in access to appropriate care and support. This study set out to explore perceptions and experiences of mental health support, including facilitators and barriers, among people with cancer to help inform national cancer policy development in Australia.

**Methods:**

We conducted semi-structured interviews with nine people diagnosed with cancer who accessed mental health support services or resources during their cancer care. Qualitative analysis using a social-ecological framework identified key themes that shaped their experience.

**Results:**

Psychosocial distress screening was identified as an important facilitator to enable access to needs-based supportive care services. There was strong advocacy for the role of peer support in helping individuals cope with emotional and psychological stressors associated with cancer care. There was a perception that disparities exist in the availability and accessibility of psycho-oncology services outside of metropolitan areas in Australia. Participants acknowledged that even if they had identified unmet supportive care needs and were referred for support, it was not always available, and that inequity exists in the supportive care services available based on cancer type, disease stage, and geographic location.

**Conclusion:**

National policy levers aimed at improving the integration and implementation of psychosocial distress screening in cancer care and addressing inequities in access to supportive care services, such as peer support and psycho-oncologists, are needed to ensure adequate support for psychological wellbeing.

**Supplementary Information:**

The online version contains supplementary material available at 10.1007/s00520-025-09593-5.

## Introduction

A cancer diagnosis and treatment are an emotionally and psychologically challenging experience that can negatively impact an individual’s mental health. This can present as psychological (depression, anxiety, and mood disorders) or emotional distress (shock, fear, and uncertainty), which can hinder acceptance of cancer treatment, quality of life, recovery, and survival [1]. It is estimated that around 50% of people diagnosed with cancer will experience psychological distress [2]. This can involve emotional, behavioural, and cognitive factors that adversely impact an ability to cope with any or all aspects of the cancer experience [3, 4]. In some instances, this distress results in psychological disorders that require specialist mental health support, including psycho-oncology. The prevalence of psychological disorders, such as depression and anxiety, is higher among people with cancer than in the general population, with estimates varying based on the treatment setting, type of cancer, and time since diagnosis [1]. This can impact an individual’s capacity to tolerate or adhere to treatment and engage in treatment decision-making [5, 6].

Many people rely on family and peers and/or support provided by non-government organisations and cancer care teams to help them cope with psychological and emotional distress [7]. Their psychological and emotional needs may change over time, and their psychosocial and supportive care needs may persist from pre-diagnosis through to survivorship [8]. Psychosocial distress screening can detect common problems that may increase the presence of distress [2]. Distress screening and referral to psychosocial or supportive care services are recommended best practices for people affected by cancer [9, 10]. Access to screening and mental health support should be a component of care for all people, regardless of their type of cancer or where they receive care.

This research aimed to explore experiences of mental health support among people affected by cancer and their perceptions of facilitators or barriers that impacted access to supportive care services. Conducting qualitative research with people with lived experience of cancer who accessed mental health support enabled a deeper understanding of how expectations and experiences shape perceptions. This also helped to contextualise individuals’ perceptions of the role of the individual, healthcare provider, and system in delivering mental health support. The findings of this study will help inform national policies aimed at improving the availability and accessibility of mental health support for people affected by cancer care in Australia.

## Materials and methods

### Conceptual framework

The social-ecological model was used to guide the analysis for this study. First devised by Bronfenbrenner [11], this theoretical framework acknowledges the complex interplay of factors in a person’s environment and represents a holistic approach to addressing public health issues [12]. The model adopted in this study consists of four levels, conceptualised as the individual, which accounts for a person’s biological and behavioural determinants; the interpersonal level, the interactions a person has with their family, friends, and peers; the organisational level, the interactions a person has with support organisations, including formal support offered by the cancer care team, and informal support provided by non-government organisations and community services; and finally, the societal level, which incorporates societal norms, regulations, and policies that affect the way people access care.

### Participant selection and recruitment strategies

**Fig. 1:**
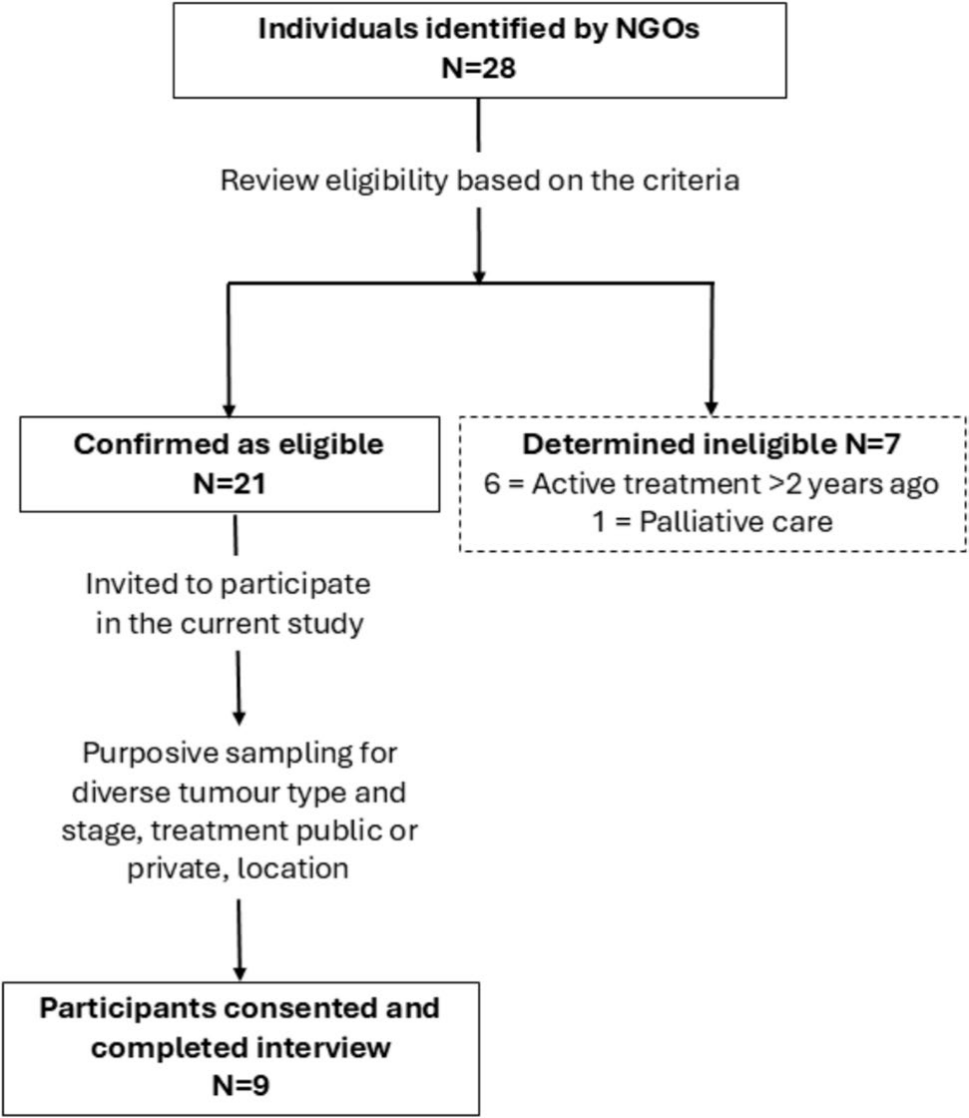
Flow diagram of participant recruitment into the study

The sample population were people diagnosed with cancer who are currently receiving active cancer treatment or within the last 2 years. The study was restricted to people who reported having accessed mental health services and resources during or after their treatment, as they had experience with the availability and accessibility of mental health support. These people were identified by non-government organisations (NGOs) who had recruited them as consumer advocates, and they expressed an interest in participating in research. Due to the proposed method of recruitment, it was expected that it would be challenging to recruit people with a terminal cancer diagnosis and/or receiving end-of-life palliative care, so it was decided they would be better served by research designed specifically to support their participation and assess their psychosocial needs. A total of 28 individuals were identified to participate; seven were subsequently deemed ineligible (see Fig. 1). We used purposive sampling to select individuals with different experiences of cancer, based on their cancer type and stage, whether they received treatment at a public or private hospital, and in different Australian states. Selected individuals were sent an email with the participant information sheet explaining the research (for more information refer to Online Resource 1) and a request to confirm their interest and eligibility by confirming in writing their consent to participate. Upon receipt of written consent, a pre-screening call was conducted to discuss the research, and verbal informed consent was obtained to participate in the interview stage. Before the interview, participants were sent a copy of the interview questions (for more information, refer to Online Resource 2). The semi-structured interview questions were reviewed by a trained psychologist (MV), to ensure they were trauma-aware and sensitive to the mental and emotional wellbeing of participants. Pre-screening calls and interviews were all conducted by the same researcher (CL). Participants were informed that the research was being conducted to inform the development of national policy by Cancer Council Australia, and that researchers were, at the time of the study, Cancer Council Australia employees. TB is an Honorary Professor at the University of Wollongong Australia, so this research was submitted to the institution’s HREC.

### Data collection

The threshold for saturation, and conclusion of recruitment, was agreed upon before data analysis, as the point at which no new barriers or facilitators were identified by the participants. Data saturation occurred after conducting nine interviews. The participants’ demographic data is provided descriptively in the “Results” section. Interviews were conducted for up to 1 h with the researcher (CL) and were recorded and transcribed via Microsoft Teams. The interviewer produced field notes to compare against the transcript to confirm it was an accurate representation of the conversation. Deidentified transcripts were analysed by another researcher (DM) using NVivo 1.7.1 software.

### Data analysis

Data analysis was completed in two phases. The first phase incorporated an inductive approach, similar to Braun and Clarke’s six steps of thematic analysis [13]. The objective was to identify key themes related to accessing mental healthcare during the diagnosis and treatment of cancer and barriers and facilitators were identified. The second phase was completed deductively, using the social-ecological model which has been previously used in cancer care-focused studies [14]. This aimed to group the barriers and facilitators identified in the first phase across the four levels of the model, which are presented narratively in the “Results” section.

## Results

### Participants

There were nine participants in total, five females and four males. Six participants lived in metropolitan areas of Australia, whereas three lived in non-metropolitan areas. Table 1 contains the demographic characteristics of the participants.

**Table 1:** Participant demographics (*n* = 9)

Characteristics	
Female	5
Male	4
Place of residence	
Metropolitan (major cities)	6
Non-metropolitan (outside major cities)	3
Treatment centre type	
Private	4
Public	5
Type of cancer	
Breast	3
Prostate	1
Neuroendocrine	2
Lymphoma	2
Adenocarcinoma	1
Disease stage	
Metastatic (non-terminal cancer)	2
Non-metastatic	7

### Thematic analysis

**Fig. 2:**
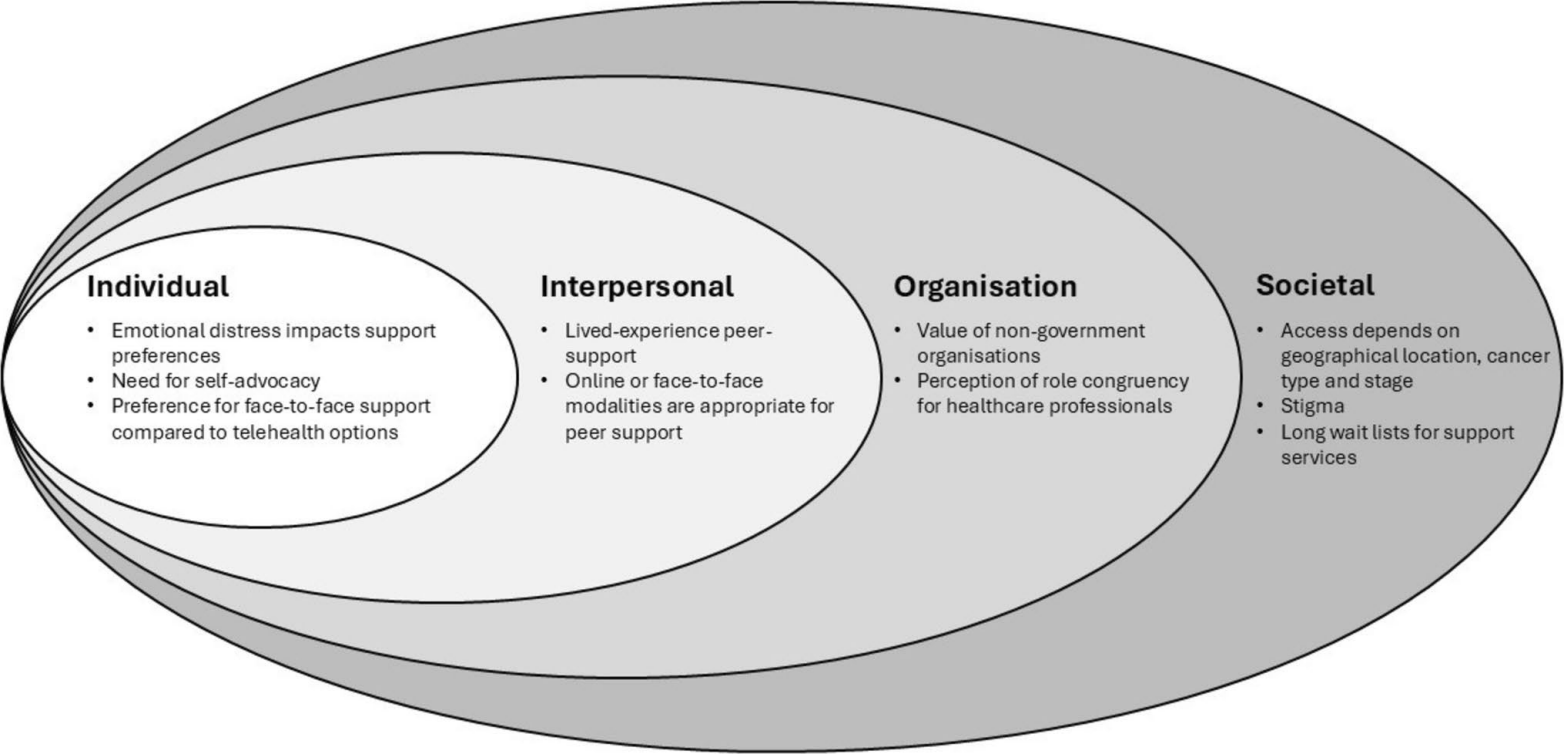
Summary of the themes explored by participants mapped onto the social-ecological model

A summary of the themes identified in this analysis can be seen in Fig. 2.

#### Individual level

The main theme at an individual level is related to the need for information, resources, and support that minimise the impact of cancer on mental health. Participants discussed how their personal experiences of emotional and psychological distress impacted their preferences for support.

Everybody has a different way of coping with these things and there isn’t a one size fits all approach. [P5]

I think there’s a fine balance between the right amount of information and too much information or not enough information. It’s a personal thing. [P7]

I didn’t need the education. I needed the hand holding, the friend, the person that’s going to just sit there and let me process. [P1]

Participants reported feeling like their mental health was not prioritised or assessed by their cancer care team. They perceived self-advocacy as important in making their needs known to their cancer care team to enable their access to supportive care or resources.

Nobody really talked in depth to me about how it might be affecting me. I wonder whether that’s a lack of training or a lack of understanding. [P3]

It’s been more me seeking out what I need when I recognise, I’m not coping. If I needed help, then I could find it. [P6]

Participants preferred mental health support that was delivered in a one-on-one face-to-face setting over group settings or telehealth arrangements. However, they acknowledged the role telehealth can play in overcoming barriers, such as location or restrictions in place during the COVID-19 pandemic.

I think having the option of in-person [mental health care], you know, especially with everything that’s been going on with COVID mental health is something that’s quite big. [P4]

#### Interpersonal level

The main theme at an interpersonal level was the importance of peer support in helping an individual cope with psychological and emotional distress and navigate supportive care services. This included organised peer support groups and/or support from family, friends, and/or carers, but it was acknowledged it was most beneficial when offered by people with lived experience of cancer.

Peer support is good because they have the same circumstances so they can express how things might turn out, should turn out, and how you should behave or react or respond to these things. [P8]

I ended up with a cancer buddy through my physiotherapist, she introduced me to one of her patients who was a bit younger, and we started having a coffee and would catch up, because she lived close to me. [P6]

My stepmum had cancer, so I had her to talk to. I had people I could talk to about it and people who had gone through the same kind of experience and share. [P6]

People living outside of metropolitan areas acknowledged that online forums for peer support was invaluable.

I’m so isolated here as I had to move home to look after my elderly mum and I don’t have any close friends. I live on Facebook Messenger. I think it’s very important to have someone to talk to. [P9]

#### Organisational level

The main theme at an organisational level was around the important role non-government organisations play in Australia in providing access to information, support, and resources.

The helplines I rang a couple of times just to have a chat with somebody offline, and not my wife, so that I could just talk about some things and lay it bare, you know, and there are always a lot of questions. [P8]

They sent me an information pack, which was handy, with self-care tips and a pamphlet on ‘what you’re feeling is kind of normal’ and ‘you’ll go through these emotions’, ‘you’ll go through the anger, sadness and shock.’ [P4]

Participants expressed uncertainty about the expectation of the cancer care team to deliver psychosocial support. There was a perception that physical health was prioritised during cancer care, with a lack of opportunities to discuss mental health, but referrals to psychosocial support were offered.

I thought the nurse at the hospital would be offering more counselling and support. But they are just there to talk about your treatment and organise scans. It’s left up to the patient to find support services, such as a psychologist or psychiatrist. [P9]

Nobody really talked in depth to me about how it might be affecting me. I wonder whether that’s a lack of training or a lack of understanding. [P3]

The oncologist was the one who provided the list of allied health providers and services. That and I ticked psychology services on the list. [P3]

#### Societal level

The main theme at a societal level was around the perception that the availability of mental health support and types of supportive care services specific to an individual’s needs would depend on their location, cancer type, and/or stage.

I joined peer support groups for a long time, about a year before I withdrew. Because I had a rare cancer, I didn’t fit into a pigeonhole, so they put me with a group for stage 4 patients with lung, breast cancer, bowel, or prostate cancer. [P7]

There were posters at the radiology and chemotherapy treatment area about information and support for wigs and makeup for breast cancer patients, but not specific to any other cancer. It made me think, there’s nothing out there, if it was out there it would be posted just like the breast cancer poster. If they had the information, I feel like they’d share it. [P1]

Participants referred to the stigma associated with mental health as a barrier to acknowledging their needs and it influenced their willingness to discuss or access supportive care.

…I also think there’s still stigma associated with mental health. People may think I’m crazy if I see a psychologist, but I just need a person to discuss things and have a chat. [P9]

Participants living in non-metropolitan areas referred to the lack of availability of local mental health providers, specifically psycho-oncologists, and long waiting lists for psycho-oncologists in public hospitals as barriers to accessing care.

I was looking for some sort of psychology counselling sessions, but I can’t afford to pay for them myself. I’ve been on a waiting list for a psychologist for nearly two years now, and I live in a regional area and I guess that makes a difference with both the cancer care and mental health treatment available. [P3]

## Discussion

This study aimed to explore the experiences and perceptions of mental health support of people affected by cancer to inform national policies to address barriers to accessing supportive care. Participants reported barriers to accessing psychosocial and mental health support across every ecological level. Individuals experience distress differently, and their perception of the impact on their mental health can change during cancer care, from diagnosis and treatment, through to survivorship [17]. In addition, individual needs and preferences for psychosocial support (i.e. information and resources, peer or psychological support, and/or counselling) may change along the cancer continuum [8, 18, 19]. As demonstrated here and in other Australian studies, barriers exist that impact the accessibility of psychosocial care, including under-detection of needs by healthcare professionals, lack of service availability, and underutilisation of services due to practical barriers such as distance, expense, and time [16, 20]. These barriers require policy responses and quality improvement initiatives to increase awareness among patients and their cancer care team about the importance of psychosocial care and how to access supportive care services [16].

Cancer care services cannot rely on patients to proactively seek supportive care, although, as highlighted by our results, people with cancer still heavily rely on self-advocacy to access mental health support. Previous research has demonstrated that psychosocial screening can improve distress detection and cancer care delivery [9, 17, 21]. Ongoing monitoring is needed to ensure timely identification of psychosocial distress and intervention to reduce the potential impact on mental health [19]. Routine screening for psychological distress and referral to supportive care services has long been recognised as best practice in cancer care in Australia and internationally [10, 22, 23]. However, despite these recommendations, there is no national data on the rates of psychological distress screening in cancer patients in Australia [24]. Studies investigating the implementation of psychosocial distress screening cite several barriers to uptake, including a lack of time and resources, training and support, low acceptability, and failure to link it to treatment and referral to supportive care services [24–26]. This highlights the urgent need for policy to address these barriers to the integration and implementation of psychosocial screening in cancer care.

Studies have shown that peer support can deliver emotional and psychosocial support beneficial to people affected by cancer [27, 28]. This study identified a preference for peer support as a psychosocial intervention to explore experiences of emotional and psychological distress and coping strategies. Although some individuals prefer the support of family, friends, and/or carers, they acknowledge the risk of negative impacts on relationships and/or carer mental health [29]. Peer support groups, organised by non-government organisations, offer individuals the opportunity to interact and engage with cancer survivors outside their immediate support network. This support can help counteract emotional and psychological distress and assist individuals in navigating supportive care services [30, 31]. Peer support is established as an important component of psychosocial care for people affected by cancer, but inequities exist in their availability for individuals with specific needs based on their cancer type and stage and geographic location.

In Australia, geography presents a significant challenge in the delivery and availability of high-quality mental healthcare services [16]. Despite widespread recognition that people affected by cancer who live outside metropolitan areas experience worse psychological distress and have limited access to psychosocial care, there are still significant disparities in mental health support across Australia [8, 16]. This study adds to the overwhelming level of evidence that people living outside of metropolitan areas experience inequity in accessibility and affordability of mental health support. This can lead to delays in accessing public psycho-oncology services or out-of-pocket costs to access private psychologists, who may lack the cancer-specific expertise and experience [32]. The higher burden of psychological needs and limited availability of mental health services has significant implications for people with cancer in these regions [16]. Improving the availability and affordability of psycho-oncology services should be an urgent priority to improve the mental healthcare of people affected by cancer to ensure they are not disadvantaged based on where they live.

Digital technology offers an opportunity to address inequities of access to psychosocial and psycho-oncology services for people living with cancer across Australia. Telehealth can help overcome barriers of distance to enable psychosocial screening and the delivery of supportive care services to people living outside metropolitan areas [33]. This is one example of how digital technology can improve access to mental health and further research is warranted to investigate if it could improve the integration of standardised psychosocial screening and supportive care into cancer care pathways to improve overall psychological wellbeing.

### Policy implications

In Australia, psychosocial and supportive care services are available to address the mental health needs of people living with and beyond cancer. However, as this population grows, the availability and accessibility of such services should be assessed, including the integration and implementation of these services and whether they are meeting the needs of people with cancer [15, 16]. Even though support may be available, the experience of participants in this study indicates that individual, organisational, and societal barriers may need to be addressed to ensure equity of access to achieve optimal outcomes. This study supports the existing evidence of the need to improve the psychosocial care of people affected by cancer and the need for policy responses to address this deficit.

Despite psychosocial and supportive care being recommended as best practice in cancer care, here we demonstrate this is not being consistently integrated and implemented, due to existing barriers. Better clinical guidance, training, and the implementation of technology to address these barriers could improve access. This will require policymakers to identify levers that can be used to improve the integration of psychosocial distress screening into clinical pathways; equity of access to supportive care, including specialist mental health services and peer support initiatives; and access to telehealth psychosocial screening and psycho-oncology services. These policy interventions could increase access to high-quality evidence-based mental health support for all Australians affected by cancer.

### Strengths and limitations

Our study benefits from several methodological strengths. The purposive recruitment of participants with different cancer experiences across metropolitan and non-metropolitan regions provided insights into mental health support during cancer care across Australia. Our analytical framework, combining inductive and deductive approaches anchored in the social-ecological model, facilitated robust theme identification across multiple levels of influence. However, several limitations warrant consideration. First, despite achieving thematic saturation, our sample size of nine participants limits the diversity of experiences captured. While qualitative research prioritises depth over breadth, additional participants might have revealed further complexities in how varying cancer journeys intersect with mental health support systems.

Our recruitment strategy introduced potential selection bias by focusing exclusively on people with a lived experience who were affiliated with NGOs. These individuals typically possess higher health literacy, enhanced navigation skills, and established connections within the healthcare ecosystem compared to the general cancer population. Their advocacy roles and organisational affiliations may have coloured their perspectives, potentially underrepresenting the barriers faced by those without similar connections and resources. Additionally, their responses might reflect positive bias toward organisations that facilitated their recruitment.

A significant limitation of our study was the deliberate inclusion of only participants who had accessed mental health services and resources. This sampling decision enabled detailed exploration of service experiences but precluded understanding the perspectives of individuals who did not access support despite potentially experiencing distress. The barriers, needs, and perceptions of non-users likely differ substantially from our sample. Furthermore, the deliberate exclusion of individuals with terminal illness represents another significant gap in our findings. The psychological support needs and service experiences of those facing end-of-life concerns differ substantially from individuals with non-terminal diagnoses. Consequently, our results may not adequately address the unique challenges and supportive care requirements for this vulnerable population, and future research should specifically target these groups to better understand barriers to access and develop more inclusive supportive care models.

A limitation was the potential impact of the COVID-19 pandemic on participants’ experiences of mental health support during cancer care in Australia. We acknowledge this may have impacted their ability to access psychosocial and supportive care services, due to increased demand, travel restrictions, and limitations on face-to-face appointments. Participants were offered alternative arrangements, including telehealth services, which were acknowledged as a suitable alternative to facilitate access for people living in non-metropolitan areas. While these circumstances provided insights into potential system adaptability, they also limit the transferability of findings to standard care conditions.

The retrospective nature of participant accounts introduces potential recall inaccuracies, despite our efforts to mitigate this by recruiting individuals diagnosed within the past 2 years. Memory distortion and cognitive reframing of past experiences could influence the narratives shared. Furthermore, participants’ current mental health status might have affected their recollection and interpretation of previous support experiences.

Future research should address these limitations through larger, more diverse samples, inclusion of individuals with terminal diagnoses, complementary methodological approaches, and triangulation with healthcare provider perspectives to develop a more comprehensive understanding of mental health support needs throughout the cancer continuum.

## Conclusions

This qualitative study provides important insights into the barriers affecting access to mental health support for people with cancer across Australia. Our findings highlight significant challenges across every level of the social-ecological model, from individual self-advocacy needs to systemic service gaps, particularly in non-metropolitan areas. Despite longstanding recommendations for routine psychological distress screening and referral pathways, implementation remains inconsistent and inadequate. The experiences of our participants underscore the critical role of peer support in addressing emotional and psychological distress, yet access to these services varies considerably based on cancer type, stage, and geographic location. Digital technologies, particularly telehealth, offer promising opportunities to bridge these gaps and improve equitable access to psychosocial care. To address these challenges, policy interventions are urgently needed to enhance the integration of psychosocial screening into standard cancer care, improve access to specialist mental health services, expand peer support initiatives, and leverage telehealth approaches. These measures are essential to ensure all Australians affected by cancer can access timely, appropriate mental health support regardless of their location or circumstances.

## Supplementary Information

Below is the link to the electronic supplementary material.

Supplementary file1 (DOCX 30.5 KB)

Supplementary file2 (DOCX 45.1 KB)

## Data Availability

No datasets were generated or analysed during the current study.
