# Supplementary material for: The need for improved integration of psychosocial and supportive care in cancer: a qualitative study of Australian patient perspectives
Source: Support Care Cancer. 2025 May 30;33(6):516. doi: 10.1007/s00520-025-09593-5 (PMC12125069; doi:10.1007/s00520-025-09593-5)
Supplement: Supplementary file 1 — Supplementary file1 (DOCX 30.5 KB) [file 520_2025_9593_MOESM1_ESM.docx]

**Title:** The need for improved integration of psychosocial and supportive care in cancer: A qualitative study of Australian patient perspectives.

**Journal:** Supportive Care in Cancer

**Authors:**

Clare Lynex PhD^1^ ORCID: 0009-0009-2533-4289

Drew Meehan MPH^1^ ORCID: 0000-0003-4768-0122

Kate Whittaker MPH^1^

Tanya Buchanan PhD^2^ ORCID: 0000-0003-3698-9351

Megan Varlow M.Psych^1^ ORCID 0009-0000-4964-3435

1. Cancer Council Australia, Sydney, Australia
2. School of Health and Society, Faculty of the Arts, Social Sciences and Humanities, University of Wollongong, Wollongong, NSW, 2522, Australia

**Author for correspondence:** Clare Lynex, Email: [clare.lynex@cancer.org.au](mailto:clare.lynex@cancer.org.au).

**Participant information sheet**

**Why have I been invited to participate in this research?**

You have been invited to participate in the Cancer Council Australia research project into mental health support, because of your lived experience with cancer care in Australia. Your participation will provide a general understanding of current gaps in the mental health care support services and resources available to people undergoing cancer care.

**Who is conducting this research?**

This research is being conducted by Cancer Council Australia.

**What does participation in this research involve?**

Participation in this research involves one pre-screening call (~15 minutes, either via videoconferencing or telephone), a recorded interview (~1 hour, either via videoconferencing or telephone) and a post-interview follow-up call (~15 minutes, either via videoconferencing or telephone). The total participation time will be 1 hour 30 minutes. The interview will focus on your experience of mental health care support services and resources you accessed during cancer care.

Participation in this research will not cost you anything. You will be compensated for your time, there is more information about this in the Remuneration Information Sheet.

**Do I have to take part in this research project?**

No. Participation in this research is voluntary. It is completely up to you whether you participate. Please note your relationship with Cancer Council will not be adversely affected should you choose not to participate in the research or choose to withdraw your consent at any time during the research.

**What are the benefits and risks of participation in this research?**

With your input, the findings from this project will provide information to support the development of Cancer Council policy and inform the development of future mental health support services and resources.

You may feel distress or discomfort in talking about your experiences. If this is the case, you may ask for the interviewer to stop at any time and withdraw your consent.

**What will happen to the information I have provided?**

If you choose to participate, then your consent will be sought for the use of the data you provided to be used for the purposes of this research project only. The data collected in this research will be stored on secure servers at Cancer Council Australia with only the Project team having access. Your data (audio recording and transcript) collected as part of the interview process will be coded so that it is only identifiable to members of the project team. This de-identified data will be stored on a secure server with access only granted to the project team. In any resulting publication and/or policy development the information you have provided will be de-identified in such a way that you cannot be identified. You may access your original interview recording at any time; however, interview transcriptions will not be accessible as records will be de-identified during the transcription process. The data that you provide will be used to inform Cancer Council policy and a publication in an open-access peer-reviewed journal, as is stated in the consent form. The results of the research will be available to you upon request.

In accordance with the relevant Australian privacy laws, you have the right to access information collected and stored by the researchers about you. This data will only be stored for 5 years. If you decide to withdraw consent or would like to access the data during this time, you contact the Project Lead to request access or if you withdraw consent for your data to be used in this research, this data will be deleted upon request.

**Who should I contact if I have questions, about this research?**

For further information about this research, please contact the Project Lead Clare Lynex at [clare.lynex@cancer.org.au](mailto:clare.lynex@cancer.org.au); or (02) 8256 4143.

**What should I do now?**

Please consider whether you would like to participate in this research. If you agree to participate, we will provide you with a consent form to complete and then follow up with you to schedule a time for the pre-screening call at a time most convenient to you.

The University of Wollongong Human Research Ethics Committee has approved this research project (Ethics protocol number 2023/250), so that it meets ethical standards and ensures the confidentiality of participants' information is protected. If you have any complaints or concerns about the way this research is being conducted, and you wish to speak to someone, you can contact (the UOW Ethics Officer, +61 2 4239 2191 or email uow-humanethics@uow.edu.au).

**What if I experience any mental health issues while participating in this research?**

We acknowledge that participating in this research may raise some uncomfortable feelings or thoughts, and you may want to talk to someone, below is a list of support services you can contact:

| **Support services** | | |
| --- | --- | --- |
| **Telephone Website** | | |
| **Cancer Council**  **Lifeline** | 13 11 20  13 11 14 | cancer.org.au  Lifeline.org.au |
| **Suicide Call Back Service** | 1300 659 467 | Suicidecallbackservice.org.au |
| **Beyond Blue** | 1300 224 636 | Beyondblue.org.au |
| **MensLine Australia** | 1300 789 978 | Mensline.org.au |
| **Other online resources** | | |
| **Head to Health** |  | Headtohealth.gov.au |
| **Life in Mind** |  | Lifeinmindaustralia.com.au |
| **SANE** |  | Saneforums.org |
