# Supplementary material for: The need for improved integration of psychosocial and supportive care in cancer: a qualitative study of Australian patient perspectives
Source: Support Care Cancer. 2025 May 30;33(6):516. doi: 10.1007/s00520-025-09593-5 (PMC12125069; doi:10.1007/s00520-025-09593-5)
Supplement: Supplementary file 2 — Supplementary file2 (DOCX 45.1 KB) [file 520_2025_9593_MOESM2_ESM.docx]

**Title:** The need for improved integration of psychosocial and supportive care in cancer: A qualitative study of Australian patient perspectives.

**Journal:** Supportive Care in Cancer

**Authors:**

Clare Lynex PhD^1^ ORCID: 0009-0009-2533-4289

Drew Meehan MPH^1^ ORCID: 0000-0003-4768-0122

Kate Whittaker MPH^1^

Tanya Buchanan PhD^2^ ORCID: 0000-0003-3698-9351

Megan Varlow M.Psych^1^ ORCID 0009-0000-4964-3435

1. Cancer Council Australia, Sydney, Australia
2. School of Health and Society, Faculty of the Arts, Social Sciences and Humanities, University of Wollongong, Wollongong, NSW, 2522, Australia

**Author for correspondence:** Clare Lynex, Email: [clare.lynex@cancer.org.au](mailto:clare.lynex@cancer.org.au).

**Interview Guide**

| **PRE-SCREENING CALL** | |
| --- | --- |
| **Introduction** | **Thank you for nominating yourself to participate in Cancer Council Australia’s Research on mental health support during cancer care.**   - The purpose of today’s call is to provide you with some information about what is involved in participating in this research. - Are you available now to speak for 15 minutes or would you like me to call you back at another time? |
| **Overview CCA and Community Reference Group** | **Firstly, I want to give you some background information on the Cancer Council Australia organisation and why we are conducting this research**   - Cancer Council is Australia’s peak non-government cancer control organisation, involved in all areas of cancer control, and advises the Australian Government and other bodies on evidence-based policies to help prevent, detect, and treat cancer. - We are developing a National Cancer Care Policy to address key areas of importance for Cancer Council nationally. For this policy chapter, we will focus on gaps in the current provision of mental health services and resources for people with lived experience of cancer. - As part of this research we are conducting interviews with people affected by cancer who would like to share their experiences of accessing mental health services and resources during cancer care. - My role, as the Project Lead is to conduct the interviews with consumers and carers and incorporate this feedback into the development of the policy chapter and manuscript for publication. - Your participation will involve a one-on-one interview with me, which will take ~1 hour and will be an opportunity to discuss your experience of mental health support services/resources during your cancer journey. - Be assured anything we discuss will remain confidential, your details and the information you provide will remain anonymous. When we use your feedback to draft policy actions and prepare a manuscript for publication it will be de-identified and aggregated with other participants feedback to ensure your privacy is maintained. Once we have compiled the draft policy and manuscript you will share a copy with you. - The expected outputs of this research project will include the chapter of the National Cancer Care Policy, a publication in an open-access peer-reviewed journal and a potential conference abstract and/or poster.   **You will have received a copy of the Participant Information sheet, which includes further details about this research.**  **Have you had a chance to review the document – Do you have any questions?**  **Can you confirm if you are comfortable in participating in an interview in English?**  **Can you confirm that you do not have any previously diagnosed learning difficulties, cognitive disorders or severe or complex mental health conditions that would prevent you from providing consent or participating in this interview?** |
| **Remuneration** | **To confirm you will receive remuneration in recognition of your time contributed to participate in this research.**   - You will have received the Remuneration Information sheet, which outlines Cancer Council’s remuneration policy. - In recognition of the important contribution, you are making by bringing your knowledge and experience you will be remunerated for time with a $100 voucher. This will be emailed to you upon completion of the research interview.   **Do you have any questions about remuneration?** |
| **Next Steps** | **Can I confirm that you consent to participate in this research and are happy to organise a time for an interview?**  **If NO,** I will then withdraw your participation in this research, thank you for your time today.  **If YES**, thank you for confirming your consent to participate. Are you available <Date, time> to schedule the 1-hour interview?   - I will send you a calendar invite with the date and time of the interview. - This will be conducted as a Teams videoconference call. Are you happy that you can use this teleconferencing platform?   **If NO,** would you prefer if I arrange the interview to be conducted as a telephone call?   - Before the interview if you have any additional questions, or want to change your mind about participating, you can contact me at any time to discuss. - Thank you for your time today. |

| **INTERVIEW** |
| --- |
| **Thank you for agreeing to participate in this research interview**  **We have 1 hour scheduled, are you happy you have sufficient time to proceed now?**   - The focus of the interview will be on your experience of mental health care support services/resources during your cancer journey. - Please note that anything we discuss will remain confidential and your feedback will be de-identified (by removing any personal or identifying information) and aggregated with that of other participants to ensure your privacy is protected in any subsequent publications. Once we have compiled the draft policy and manuscript for publication, we will share this with you. - If at any point during the interview, you like to pause or end the conversation, please let me know. You are free to discontinue your participation at any time without providing any reason. - If after today’s interview, you do experience any emotional distress, as a result of what we have discussed, the Participant Information Sheet has details of mental health support services you can contact.   **Did you have any questions about the information in the Participant Information or Remuneration Information sheet?**  **Can I confirm that you consent to participate in this interview and for it to be recorded?**  **If NO,** I will then withdraw your participation in this research, thank you for your time today.  **If YES, I would like to ask if you could start by sharing with me your experience of mental health issues you experienced during your cancer journey?** |
| **Follow-up questions**  **If the following is NOT covered in the initial discussion try to cover each as it relates to their individual experience**   - Thinking back on this experience, what mental health support services/resources did you access? - How and when did you find out about these support services/resources? - What advice would you give someone else who is now in a similar situation about how to find out about and access mental health support? - Are there any suggestions you’d like to make to healthcare services, hospitals, or government decision-makers about what would be helpful for people like you in this situation? |
| **Cancer Diagnosis**   - Thinking back to when you were first diagnosed, were there services/resources that you know about now, that could have helped you? - What aspects of the support services/resources you did access did you find helpful/unhelpful? - What would you like to see as the ‘standard of care’ for anyone newly diagnosed with cancer to support their mental health? - Did you undergo any kind of mental health needs assessment at diagnosis, or at any point during your cancer journey? - **If yes:** How frequent were the assessments (specific time points)? - Do you think these assessments adequately captured your concerns and needs? - How did your treating team respond to and use these assessments? - What services/resources did you receive as a result of these assessments? |
| **Cancer Journey**   - Did you seek out access to psychosocial or psycho-oncology support services (i.e. psychologist, social worker, counsellor, psychiatrist, peer-to-peer, support groups)? - How did you become aware of these services? - Can you describe any specific aspects that were helpful or unhelpful? - If you could recommend three changes to the way the mental health needs of people affected by cancer are supported, what would they be and why? |
| **Survivorship**   - If you have completed active treatment, since you have completed your cancer care would you say your mental health has improved or worsened?   Do you feel you were adequately supported after discharge from the cancer care team? |

| **Closing and questions** | **Thank you for taking the time to speak to me today.**   - I really appreciate your willingness to share your story and experience of mental health and cancer care, this has been valuable in informing and shaping our cancer care policy. - We will send you a follow-up email to confirm the next steps in the process, but you can email or call me at any time if you would like more information or have any questions. - I will give you a follow-up call tomorrow to check to see if you need any additional support.   **Is there anything else you would like to add or ask about what we have covered today?** |
| --- | --- |

| **POST-INTERVIEW CALL** | |
| --- | --- |
| **Introduction** | **Thank you again for your participation in the Cancer Council Australia’s Research on mental health support during cancer care.**   - The purpose of today’s call is to provide you with some information about what is involved in participating in this research. - Are you available now to speak for 15 minutes or would you like me to call you back at another time? - Are you happy that you got what you wanted out of the interview – is there further feedback you would like to provide? |
| **Remuneration** | **To confirm you will receive remuneration in recognition of your time contributed to participate in this research.**   - You will have received the Remuneration Information sheet, which outlines Cancer Council’s remuneration policy. - In recognition of the important contribution, you are making by bringing your knowledge and experience you will be remunerated for time with a $100 voucher. - After today’s call I will send you an email with the voucher attached.   **Do you have any questions about remuneration?** |
| **Next Steps** | - Once we have completed all the participant interviews and have compiled the feedback, I will be in touch regarding the timeline for you to receive a copy of the draft policy and manuscript for publication. - Thank you for your time today. |
